# Lessons Learned From Implementing a Culturally Tailored Virtual Support Program for Asian American Breast Cancer Survivors With Pain and Depression

**DOI:** 10.2196/81187

**Published:** 2026-06-03

**Authors:** Seulgi Ryu, Dongmi Kim, Wonshik Chee, Eun-Ok Im

**Affiliations:** 1 School of Nursing, The University of Texas at Austin, 1710 Red River St, Austin, TX, 78722, United States, 1 5124717913; 2 College of Education, The University of Texas at Austin, Austin, TX, United States

**Keywords:** breast cancer, Asian American, technology-based intervention, pain, depressive symptoms

## Abstract

Breast cancer is the most diagnosed cancer among women in the United States, with a greater increase in incidence among Asian American women than among others. Despite having resided in the United States for decades, Asian American breast cancer survivors face unique cultural challenges, including stigma and reluctance to disclose their illness. We present a Viewpoint to discuss practical issues encountered in implementing a culturally tailored, technology-based program aimed at reducing pain and depressive symptoms in Asian American breast cancer survivors. This Viewpoint is a simple ancillary content analysis of meeting minutes and research diaries from a parent study of cancer pain management; here, we identify themes related to practical issues in conducting a culturally tailored, technology-based intervention among Asian American breast cancer survivors with pain and depressive symptoms. Key issues identified include (1) reluctance to disclose, (2) variability in engagement based on disease status, (3) the need for personalized support, and (4) intraethnic differences related to various factors. Suggestions for future research include (1) tailoring communication to participants’ preferences while building trust over time, (2) applying flexible care strategies, (3) assessing individual needs early and adapting materials based on feedback, (4) matching participants with interventionists according to language and level of acculturation, and (5) respecting differences in cultural identities between ethnic subgroups. Addressing these challenges can improve the effectiveness of technology-based interventions for racial or ethnic minority groups.

## Introduction

Breast cancer remains the most commonly diagnosed cancer among women in the United States, with approximately 1 in 8 women (13%) expected to be diagnosed with breast cancer in their lifetime [1]. From 2012 to 2021, the incidence of breast cancer increased by 1% annually, with a notably steeper rise of 2.6% among Asian American women than among White, Black, and American Indian/Alaska Native women (1%) and among Hispanic women (1.6%) [1]. This increase among Asian American women coincided with the rapid growth of the Asian American population, which is projected to double between 2016 and 2060, primarily due to migration [2].

Among Asian American subgroups, breast cancer is most prevalent among Chinese (32%), Japanese (35%), and Korean (32%) American women [2]. Higher breast cancer rates are found among Asian immigrants than among US-born Asian people [3], indicating the role of immigration-related factors in health outcomes.

Despite long-term residence in the United States, Asian Americans often retain strong cultural values that shape their health care experiences. Many breast cancer survivors within Asian American communities report stigma, emotional suppression, and reluctance to disclose illness, owing to cultural expectations. Cultural factors and expectations have a significant role in gender-specific cancers. This is especially clear in many Asian cultures, where discussions relating to reproductive cancers, such as breast cancer, are often considered taboo or sensitive [4]. Furthermore, in the collective, family-centered nature of decision-making in Asian societies, women may delay or avoid care due to expectations of modesty, composure, and harmony, and may place greater emphasis on family responsibilities rather than on seeking care for themselves. This cultural factor also reflects discomfort in seeking medical attention and help because of reluctance to reveal their cancer diagnosis [5,6]. Chinese Americans report feelings of social rejection [7], Korean Americans describe fear of disclosure [8,9], and Japanese Americans often avoid seeking help altogether because of beliefs about enduring hardship alone [10]. The burden of fulfilling multiple caregiving roles as mothers, wives, and daughters intensifies this emotional toll [9,11].

Cultural stigma surrounding illness often leads to concealing the side effects of treatments, such as alopecia [8], or even refusing chemotherapy to avoid looking ill [12]. These behaviors contribute to lower levels of emotional support and reduced self-care among Asian American breast cancer survivors [11]. Language barriers and unfamiliarity with the US health care system further compound such challenges [13,14]. In navigating care, recent immigrant Asian Americans often report a lack of accessible, culturally appropriate information [11,15]. Cultural stigma around cancer pain also hinders open communication with providers, limiting access to appropriate treatment [16]. A systematic review has shown that many Asian survivors normalize pain and illness, leading to the underutilization of pain management and worsening of symptoms [17].

**Figure 1.**
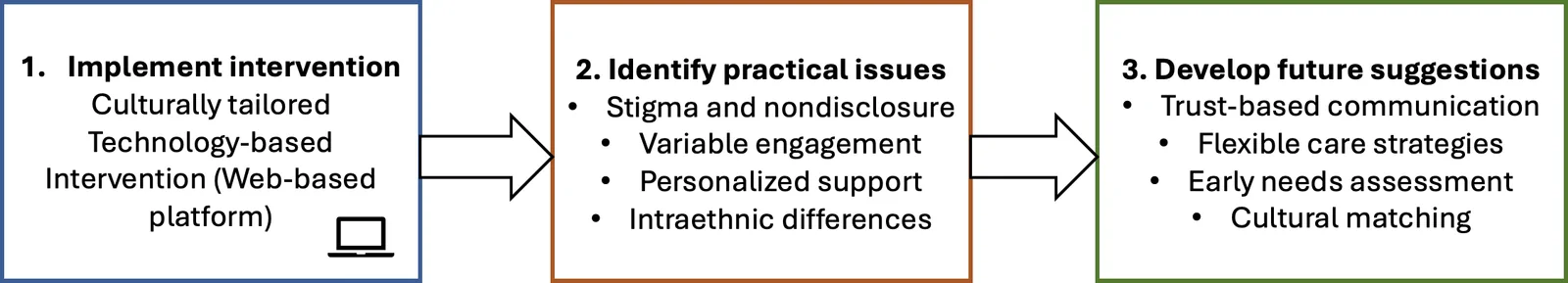
Operational framework: from implementation challenges to culturally tailored strategies.

To address these challenges, culturally and linguistically tailored interventions are increasingly emphasized [9,18]. Individuals’ cultural norms significantly influence how they perceive and respond to pain, reinforcing the importance of culturally tailored strategies to address pain and provide emotional support [19]. However, despite the importance of pain and depressive symptoms in cancer care [8,11], few studies have addressed these issues within culturally tailored programs for Asian American breast cancer survivors [12]. In response, the research team has developed a culturally tailored, technology-based web application intervention for Chinese, Korean, and Japanese American breast cancer survivors. This program focuses on reducing pain and depressive symptoms. However, multiple challenges have emerged during implementation, and the operational framework is presented in Figure 1. This Viewpoint contributes to exploring issues encountered in implementing a culturally tailored, technology-based intervention for Asian American women with breast cancer and to providing recommendations that can inform future research and support research teams in planning, implementing, and evaluating technology-based intervention studies among racial or ethnic minorities.

## The Parent Study

Our ongoing parent study is a randomized controlled trial designed to improve pain and depressive symptoms among Chinese, Japanese, and Korean American breast cancer survivors in the United States. The 3 ethnic groups were selected because they have the highest prevalence rates of breast cancer among Asian women in the United States [2]. Participants were recruited nationwide, and enrolled participants were randomly assigned to either an intervention or a control group. The intervention group received a web-based coaching and support program with a bilingual interventionist of the same racial/ethnic background through weekly chat sessions, focusing on both pain and depressive symptom management for 12 weeks. The control group received the same web-based one-to-one format, with a primary focus on pain management. Importantly, the assigned interventionists identified themselves by their professional roles (eg, registered nurse or nurse practitioner) and provided support as trained members of the research team. Furthermore, all participants received a Fitbit (Google LLC) to encourage physical activity and symptom management.

Outcomes included cancer pain management and pain experiences, including self-reported pain; digital biomarkers (eg, heart rate); accompanying symptoms, such as depressive symptoms; and quality of life. Assessments were conducted at 3 points (pretest, 1 month posttest, and 3 months posttest) during the 3-month intervention period. The study was grounded in Bandura’s social cognitive theory [20] and approved by the institutional review board of the University of Texas at Austin (parent study title: “Cancer Pain Management: A Technology-Based Intervention for Asian American Breast Cancer Survivors”; STUDY00004807).

The technology-based activities consist of three components culturally tailored for Asian American breast cancer survivors: (1) a study website providing online forums for group support where participants share their opinions and experiences based on the selected topics from the educational modules provided, and individual one-to-one coaching with an assigned interventionist; (2) online education modules available in Chinese (simplified and traditional), English, Japanese, and Korean based on topics related to breast cancer care and treatments; and (3) links to verified online sources, including the Centers for Disease Control and Prevention website. Online forums and chatting sessions were administered by bilingual interventionists, and educational modules included both general and culture-specific content for respective subethnic groups. The online resources include information from general and subethnic-specific health organizations and institutes. Anonymity was strictly protected throughout all online forums and chat sessions. All coaching and support interactions were conducted one-on-one to ensure participants had no access to other participants’ personal information. Forum participation was limited to participants of the same racial/ethnic background and was organized into small groups of approximately 10 participants, thereby further supporting privacy and a sense of safety. Additionally, all participants used self-selected nicknames, and no identifying information was visible to other participants.

## Analysis

Our research team analyzed meeting minutes and research diaries from the parent study to identify themes related to practical issues in conducting a culturally tailored, technology-based intervention among Asian American breast cancer survivors with pain and depressive symptoms. The research memos were based on data from 51 participants (n=12, 23.5% Japanese, n=17, 33.3% Chinese, and n=22, 43.1% Korean) with an average age of 50.51 (SD 9.24; range 32‐72) years. Of the participants, 12 (23.5%) communicated in English and 39 (76.5%) communicated in their own language. The average number of years spent in the United States among the 46 participants not born in the United States was 19.89 years. Only 1 (2%) participant, from the Chinese group, dropped out during the 1-year data collection period. Overall, 98% (50/51) of enrolled participants completed the intervention. Adherence to the individual coaching and support sessions was high, and intervention sessions were completed as scheduled. The research team members who conducted interventions with participants were registered nurses or nurse practitioners in the United States with clinical experience for patients with cancer for a range of years. Most of them held either a master’s degree in nursing or a doctoral degree, or were doctoral students in nursing. This analysis was performed with a simple content analysis [21]. Using Microsoft Teams, the team held weekly discussions to address challenges and maintained records of online communications, such as meeting minutes. The research team communicated weekly for 1 year (15‐16 memos per week × 4 weeks per month × 12 months=720 memos).

Research memos from weekly meeting minutes included issues raised during the intervention phases. The data included topics such as passive or active involvement; participants’ treatment and side effects, including pain or depressive symptoms; how interventionists assisted them or emotional support provided; technical issues in navigating the website and Fitbit; and participants’ reflections. Two authors with experience in qualitative coding independently reviewed and coded the data line by line. Differences were discussed, and the codes were refined to reflect their intended meanings. The themes were finalized through multiple group meetings with the research interventionists. Ultimately, categories representing practical issues in implementing coaching and support sessions were established on the basis of themes extracted from the codes. The interventionists’ research memos, which describe their perspectives and share matched themes, are presented in Table 1.

**Table 1.** Issues and interventionist perspective quotes.

Issues	Interventionist perspectives (paraphrased)
Reluctance to disclose	The participant initially showed a passive attitude, responding to questions with only “yes” or “no.” In accordance with the intervention protocol, I attempted to build rapport by asking questions drawn from the baseline survey. [J^a^] At the beginning of the sessions, the participant preferred to be asked essential questions (eg, pain or depressive symptom management, physical activity) rather than sharing details about daily life or other concerns. [K^b^] This participant did not proactively share their emotions or any pain or depressive symptoms, so I initiated the conversation by asking how their day had been and what their mood was like that week. After 3‐5 sessions, the participant became more active and open. [C^c^]
Variability in engagement based on disease status	This participant has been a cancer survivor for over 10 years, so she had relatively fewer depressive symptoms, pain, or discomfort. I provided information on a healthy diet and exercise to maintain her condition. [K] I encountered a participant who showed great appreciation for the chat sessions for providing useful information to improve side effects and sleep quality. [C and J]
Need for personalized support	We discussed the side effects of hormone therapy due to her chronic fatigue and aches, and we will discuss bone density next week, as the participant is concerned about osteopenia. [K] The participant underwent extreme mood fluctuations due to premenstrual symptoms after hormone therapy, so I provided support for her emotional health. [C and K]
Intraethnic differences related to various factors	I have noticed that English-speaking Chinese are more fitted into the US mainstream culture and understand the medical terms and how health care systems work better than non-English-speaking Chinese with limited English proficiency. On the other hand, I found that some Chinese-speaking participants had difficulty accessing medical care, so as an interventionist, I provided guidance on navigating the US health care system. [C] This English-speaking participant focused more on themselves rather than the other non-English-speaking participants who mostly centered on family care [C and K] To reduce the pain, this participant used sauna, cupping therapy, acupuncture, or patches that she shipped from Korea. [K]

a J: Japanese interventionist.

b K: Korean interventionist.

c C: Chinese interventionist.

## Observations

### Reluctance to Disclose

Three key challenges emerged during the implementation of culturally tailored individual coaching and support sessions delivered by culturally matched interventionists. First, several participants demonstrated a passive communication style during their interactions with interventionists. Often reluctant to express emotions, they frequently responded to questions with minimal engagement, offering brief replies such as “yes” or “no.” This behavior aligns with findings in previous literature, which indicate that Asian Americans tend to express emotions passively and to view not bothering others as a culturally virtuous action [11,18].

Second, some participants reported discomfort with the small talk that interventionists adopted to establish rapport [22]. These participants preferred to be asked only essential questions, thereby minimizing their participation in chat sessions. These communication patterns presented challenges for the interventionists, particularly in navigating conversations after addressing key concerns identified in the baseline survey.

Third, interventionists occasionally encountered participants who were highly proactive in managing their own well-being but reluctant to disclose specific details about their difficulties and especially cautious about sharing personal hardships. This is a common phenomenon among Chinese, Korean, and Japanese American participants, and building rapport with participants in this study required an average of 2 to 3 or 4 to 5 out of 12 sessions. Indeed, Asian American breast cancer survivors are often not open to sharing their distress, even with their physicians [9,18]. Consequently, despite the anonymity and non-face-to-face nature of coaching sessions that we expected to enhance openness, the research team faced significant barriers to establishing effective interactions with participants.

### Variability in Engagement Based on Disease Status

Participants’ responses to the culturally tailored intervention varied substantially by disease status. Individuals who were not currently experiencing significant pain or depressive symptoms, were not undergoing active treatment, had received a noninvasive breast cancer diagnosis, or had low scores on the Patient Health Questionnaire-9 (which was administered prior to the chatting sessions) [23] generally demonstrated lower levels of engagement. Many of these participants reported fewer unmet needs related to their cancer experiences and thus participated more passively during the chat sessions. Likewise, participants who had completed surgery or primary treatment more than 10 years ago often reported minimal lingering pain or symptoms [24] and exhibited low involvement with the interventionists.

In contrast, participants who were newly diagnosed or actively undergoing treatment were significantly more engaged. They frequently sought timely information, emotional support, and strategies to manage symptoms associated with their diagnosis [18]. Similarly, participants who reported ongoing bodily pain or unresolved physical discomfort were also highly proactive during the chat sessions, often steering the conversations to explore effective pain management strategies. Others with poor sleep quality or heightened anxiety related to fear of cancer recurrence demonstrated strong engagement, initiating conversations and maintaining a high level of interaction during the chat sessions [25].

### Necessity for Personalized Support

Our research protocol requires participants to complete weekly mandatory chatting sessions with culturally matched interventionists for 12 weeks. Following these sessions, participants are encouraged to engage in self-directed learning using educational modules and online resources provided by the research team. However, considerable variability was observed in participants’ engagement with these materials, which appeared to be influenced by individual differences in baseline knowledge, personal needs, and interest levels.

In response, the research team has continually revised and updated the educational content to better accommodate participants’ evolving and diverse needs. These personalized efforts are also integrated into the chatting sessions, allowing interventionists to deliver targeted, relevant information. For instance, one participant undergoing tamoxifen treatment reported experiencing severe premenstrual symptoms, which included anxiety, depressive mood, and extreme mood swings [26]. In this case, the interventionist provided tailored information addressing the definition of premenstrual symptoms, diagnostic criteria, and self-assessment methods. This individualized support not only enabled the participant to objectively recognize her symptoms but also empowered her to communicate more effectively with her health care provider to pursue appropriate treatment. Another example involved a participant undergoing active hormone therapy who was experiencing poor sleep quality. That participant focused her chatting sessions on discussing treatment-related side effects and tracking her sleep patterns using a Fitbit device provided by the research team.

### Intraethnic Differences

Two key challenges were identified in implementing our culturally tailored interventions: differences in engagement between English-speaking and non-English-speaking participants, and differences in acculturation levels. Intraethnic differences in engagement were prominent between English-speaking and non-English-speaking Chinese, Korean, and Japanese participants. English-speaking participants were generally more acculturated and exhibited less stigma regarding the disclosure of symptoms and concerns related to their cancer diagnosis [27]. They tended to be more approachable and proactive in their interactions with interventionists and frequently took the lead in conversations. Many openly shared personal information and actively sought both medical and emotional support, which may reflect cultural norms shaped by their greater integration into mainstream American society [27]. This pattern was also observed during the research team’s community outreach, in which English-speaking Chinese Americans were more willing to discuss the study than Chinese-speaking Chinese Americans. The interventionists observed that English-speaking participants demonstrated a stronger grasp of medical terminology and a clearer understanding of how the US health care system operates. They also tended to prioritize their individual well-being [28] over traditional familial roles, such as mother, wife, or daughter [11], and actively sought ways to manage their symptoms rather than silently endure them [10].

In contrast, non-English-speaking participants often encountered challenges in accessing medical care [11], in part because of limited English proficiency. These individuals required additional guidance from interventionists to navigate available health care resources [12]. Among the Korean-speaking participants, for example, role conflicts emerged frequently. These individuals, balancing the multiple responsibilities of being mothers, wives, daughters-in-law, and breast cancer survivors, reported significant internal struggles [12]. Many endured their pain quietly and were hesitant to share their difficulties with family members [11], reflecting culturally embedded values such as self-sacrifice and emotional stoicism [12].

Differences in participant engagement across acculturation levels were also observed. Participants who had immigrated more than 20 years ago or were born in the United States were generally more open to sharing personal experiences, demonstrated greater confidence in expressing their symptoms, and actively sought health-related information [29]. Some of those born in the United States adopted a hybrid approach to symptom management. For example, Chinese American participants frequently combined Western medical practices, such as the use of painkillers, with traditional remedies, such as acupuncture and herbal medicine [30]. Similarly, some Korean American participants reported using culturally familiar complementary methods, such as sauna therapy, to alleviate bodily pain, improve circulation in their swollen arm due to breast cancer surgery, and enhance overall comfort [31]. These practices, rooted in the Korean wellness tradition, were often used alongside conventional medical treatment, reflecting a culturally embedded approach to symptom management. This tendency may be attributed to the continued influence of cultural values transmitted through family, even among English-speaking, American-born individuals [32].

Conversely, recent immigrants were typically less expressive about their emotional and physical challenges and tended to rely more heavily on traditional remedies to manage their pain or depressive symptoms. They also experienced greater difficulties in adjusting to the health care system and identifying culturally appropriate services in the United States. These individuals required more intensive guidance and culturally sensitive support from interventionists, including assistance in identifying culturally relevant support groups within their communities [11,13].

We also found differences in participant engagement across other background characteristics. Although participants may belong to the same broad ethnic category, differences in political identity, language, and social norms were observed between Taiwanese and mainland Chinese participants. Some Taiwanese participants expressed discomfort with being labeled as “Chinese” and rejected this identification, emphasizing the importance of acknowledging their distinct cultural and national identities. This finding indicated the need for a more nuanced approach in implementing the study.

## Future Directions

These viewpoints have identified four practical issues in implementing a culturally tailored, technology-based program among Asian American breast cancer survivors: (1) reluctance to disclose, (2) variability in engagement based on disease status, (3) the necessity for personalized support, and (4) intraethnic differences in engagement related to multiple factors. These issues reflect the program’s culturally tailored, non-face-to-face, technology-based design. These viewpoints call for the following recommendations for such research with racial or ethnic minorities in the future, especially for research with Asian American breast cancer survivors (Table 2).

**Table 2.** Practical issues and related suggestions for future research.

Practical issues	Suggestions
Reluctance to disclose	Strive to accommodate participants’ communication preferences Understand that building trust with Asian American breast cancer survivors may require sustained effort over time Take a more proactive stance in providing emotional support and offering relevant information
Variability in engagement based on disease status	Adopt and implement flexible communication and care strategies
Necessity for personalized support	Perform a thorough initial assessment to gain a comprehensive understanding of participants Regularly update and diversify materials based on feedback from participants
Intraethnic differences	Match participants with interventionists who have similar linguistic backgrounds and levels of acculturation Address cultural identity sensitivities within each ethnic subgroup

First, interventionists should be trained to effectively support participants who may not engage interactively during chat sessions. Communication strategies can be tailored to participants’ preferences, for example, by reducing the duration of chatting sessions and fostering a supportive environment that encourages openness at participants’ own pace [33]. Beginning each session with simple, casual questions, such as “How are you feeling today?” or “How has your day been so far?” can help participants enter the conversation and reduce the potential pressure or discomfort associated with more direct inquiries.

For participants who demonstrate a passive communication style, interventionists can adopt a more proactive stance [33]. This may involve providing emotional support and gently introducing relevant information about treatment options, symptom management, and potential side effects [18]. It is crucial to recognize that trust-building with Asian American breast cancer survivors often requires sustained effort over time [34]. Therefore, interventionists should anticipate that it may take at least 2 to 3 sessions, and possibly up to 4 or 5, before participants are sufficiently open to share personal experiences or express emotional concerns in a web-based research study, according to the study’s findings. Research teams should also emphasize the advantages of anonymous, non-face-to-face interventions, especially their flexibility regarding time, location, and perceived safety from stigma.

Although Asian American breast cancer survivors are often reluctant to express emotions openly because of cultural norms of emotional restraint [7], interventionists can still foster trust and establish meaningful therapeutic relationships by attending to participants’ unmet emotional needs [18]. Prior research has shown that while many Asian American breast cancer survivors internalize feelings of depression or anxiety, they also maintain a strong desire for emotional support [9,18]. Interventionists can play a vital role in enabling participants to develop effective coping strategies by gently encouraging them to share their feelings or experiences [9].

Second, given the variability in engagement based on disease status, interventionists need to adopt and apply flexible communication and care strategies. For participants who are newly diagnosed or undergoing active treatment, it is essential to prioritize discussions of treatment-related side effects, emotional distress, and associated concerns, and to provide in-depth, individualized coaching and coping strategies [35,36]. Interventionists can support these individuals through timely guidance offered in supportive, open, interactive chat sessions that directly address their current problems and lived experiences.

In contrast, breast cancer survivors who are asymptomatic or no longer undergoing active treatment may require a different approach. These individuals may not always vocalize their physical pain or psychological distress [37]. They may also be unaware of potential long-term side effects or may continue to experience residual symptoms, such as pain [12,24]. Among long-term breast cancer survivors, fatigue is common, so interventionists should be particularly attentive to identifying and addressing such symptoms [38,39]. For these participants, interventionists may focus more on general well-being, self-management, and psychosocial adjustment to survivorship [40]. Interventionists should also remain attentive to subtle cues because even those who report no current physical or emotional symptoms may still have unspoken concerns, such as fear of recurrence, uncertainty, body change, or identity loss [40,41]. Fostering a supportive, participant-centered environment during chat sessions is essential for addressing both explicit and implicit needs.

Third, we recommend enhancing personalization by conducting a thorough initial assessment to ascertain each participant’s baseline knowledge, personal needs, and interests [42]. Our research team has provided tailored support to address participants’ specific concerns, particularly those related to treatment side effects. This has included providing detailed information on alleviating symptoms, such as bodily aches or pain resulting from hormone therapy [7]; addressing medication-related side effects, such as osteoporosis [43]; guiding lifestyle modifications; providing reliable sources for symptom relief; and offering dietary recommendations and psychosocial support [35]. Regularly updating and diversifying educational content in response to participants’ feedback and emerging needs can help ensure that learning remains engaging and addresses individual concerns [44]. A robust feedback system is also essential for gathering insights into the perceived effectiveness of educational materials and for identifying a broad range of participants’ needs [44]. This feedback can inform continuous improvements, making the intervention more supportive and meaningful. By implementing these suggestions, researchers can create a more engaging, supportive intervention environment, ultimately improving health outcomes and participants’ satisfaction.

Fourth, to accommodate the diverse needs arising from variations in language proficiency and levels of acculturation, participants should be matched with interventionists who share similar cultural and linguistic backgrounds [14]. This will facilitate greater mutual understanding and more effective communication and create a more supportive environment [13]. To account for participants’ cultural backgrounds and English-language proficiency, our research team matched first-generation participants with interventionists who had substantial knowledge of participants’ native cultural norms and lived experiences, or who had immigrated relatively recently. In contrast, second-generation participants who were more comfortable with speaking English were paired with interventionists who demonstrated a stronger understanding of US culture, had prior experience with American health care settings, and were more familiar with navigating the US health care system [45]. Overall, interventionists with more years of relevant experience were prioritized during matching to enhance the quality of interaction and cultural understanding. To ensure the quality of the matching, all interventionists, regardless of their length of stay in the United States, received standardized training and education prior to the intervention. The materials covered included US health care systems; manuals for the intervention and the forum; guidance for weekly sessions; and evidence-based educational materials to inform participants about treatments, cancer care, and culturally tailored information on pain and depressive symptom management. Furthermore, throughout the regular meetings, we shared our concerns and were consistently supervised by the principal investigators. As the study progressed, our matching strategy appeared to be effective, contributing to a more inclusive and culturally and linguistically appropriate environment for participants. Therefore, we recommend continual monitoring and evaluation of the outcomes of such detailed matching. Tracking indicators such as engagement levels, satisfaction, and perceived support can help researchers refine the matching strategy to further enhance the intervention’s overall effectiveness.

Furthermore, given ethnic subgroups’ sensitivity regarding cultural identity, participants should be matched with interventionists who share not only the same ethnicity but also similar cultural and linguistic backgrounds, even within broader ethnic categories. To address these concerns, our research team implemented strategies to ensure culturally matched and individualized coaching and support sessions. For example, Taiwanese participants were matched with interventionists of Taiwanese background; Chinese participants who had immigrated from mainland China or were US-born individuals with parents from mainland China, and who used simplified Chinese, were paired with interventionists who shared similar cultural experiences and language use [46]. Even during recruitment, the team developed both Traditional Chinese and Simplified Chinese flyers, following recommendations from Taiwanese interventionists, to appropriately reflect the linguistic and cultural differences between the Taiwanese and Chinese communities. This fostered a respectful, inclusive environment, ultimately enhancing the intervention’s effectiveness.

In the era of proliferating artificial intelligence (AI)–assisted tools for cancer care, AI-powered chatbots have benefited patients with breast cancer by being available 24/7, given the unpredictability of the disease and associated anxiety, and by offering interactive, personalized assistance with easy access [47]. To accommodate the suggestions of this research, AI chatbots could be integrated with human interventionists as a powerful tool for meeting individual needs or for helping individuals become more engaged, especially when stigma, emotional suppression, or reluctance to disclose breast cancer due to cultural norms may hinder help-seeking. Furthermore, they could provide tailored information to address individual needs, offer more dynamic, interactive environments for breast cancer survivors, and potentially support emotional well-being [47,48]. However, concerns have been raised about AI chatbots’ ability to understand human emotions [49]. Human touch and interpersonal interactions could therefore complement AI chatbots, helping breast cancer survivors feel genuinely cared for. Furthermore, future studies need to investigate incorporating features for language support, including cultural nuances and even dialects into AI-powered chatbots to provide culturally tailored supportive care for Asian American breast cancer survivors.

## Conclusions

On the basis of an ongoing study with Asian American breast cancer survivors, this Viewpoint discusses practical challenges in implementing a culturally tailored, technology-based web application program among Asian American breast cancer survivors with pain and depressive symptoms, and we provide several recommendations for such research in the future. We offer these recommendations to inform the planning and implementation of future culturally tailored, technology-based interventions among racial or ethnic minority groups, specifically Asian American breast cancer survivors.

## Abbreviations

AI: artificial intelligence
