# Correction: Lessons Learned From Implementing a Culturally Tailored Virtual Support Program for Asian American Breast Cancer Survivors With Pain and Depression

**DOI:** 10.2196/81186

**Published:** 2026-09-25

**Authors:** Seulgi Ryu, Dongmi Kim, Wonshik Chee, Eun-Ok Im

**Affiliations:** 1 School of Nursing, The University of Texas at Austin, 1710 Red River St, Austin, TX, 78722, United States, 1 5124717913; 2 College of Education, The University of Texas at Austin, Austin, TX, United States

In “Lessons Learned From Implementing a Culturally Tailored Virtual Support Program for Asian American Breast Cancer Survivors With Pain and Depression” [1], the authors noted one error.

The funding information has been revised from the following:

Research reported in this publication was supported by the National Cancer Institute of the National Institutes of Health (grant R33CA280979). The content is solely the responsibility of the authors and does not necessarily represent the official views of the National Institutes of Health.

The Funding section now reads:

Research reported in this publication was supported by the National Cancer Institute (NCI) and the National Institute of Neurological Disorders and Stroke (NINDS) of the National Institutes of Health through the NIH HEAL Initiative under award R33CA280979. The content is solely the responsibility of the authors and does not necessarily represent the official views of the National Institutes of Health.

The correction will appear in the online version of the paper on the JMIR Publications website, together with the publication of this correction notice. Because this was made after submission to PubMed, PubMed Central, and other full-text repositories, the corrected article has also been resubmitted to those repositories.
